# An Update on the Exploratory Use of Curcumin in Neuropsychiatric Disorders

**DOI:** 10.3390/antiox11020353

**Published:** 2022-02-10

**Authors:** Nicolás Lamanna-Rama, Diego Romero-Miguel, Manuel Desco, Maria Luisa Soto-Montenegro

**Affiliations:** 1 Instituto de Investigación Sanitaria Gregorio Marañón, 28007 Madrid, Spain; nlamanna@hggm.es (N.L.-R.); ldromero@hggm.es (D.R.-M.); 2 Departamento de Bioingeniería e Ingeniería Aeroespacial, Universidad Carlos III de Madrid, 28911 Madrid, Spain; 3 Centro de Investigación Biomédica en Red de Salud Mental (CIBERSAM), 28029 Madrid, Spain; 4 Centro Nacional de Investigaciones Cardiovasculares (CNIC), 28029 Madrid, Spain; 5 High Performance Research Group in Physiopathology and Pharmacology of the Digestive System (NeuGut), Universidad Rey Juan Carlos, Alcorcón, 28922 Madrid, Spain

**Keywords:** curcumin, psychiatric disorders, inflammation, oxidative stress, schizophrenia, autism, depression, Obsessive Compulsive Disorder

## Abstract

Curcumin is a polyphenol extracted from the rhizome of the turmeric plant. Beyond its common use as a culinary spice in Eastern Asia, curcumin has been proposed as a therapeutic compound due to its antioxidant, anti-inflammatory and neuroprotective properties. Thus, its efficacy has been evaluated in various inflammatory-based psychiatric disorders, such as schizophrenia, depression, or autism. Our aim is to review those preclinical and clinical studies carried out in psychiatric disorders whose therapeutic approach has involved the use of curcumin and, therefore, to discern the possible positive effect of curcumin in these disorders. Preclinical studies and completed clinical trials of curcumin for psychiatric disorders published from January 2005 to October 2021 were identified through searching relevant databases until 31st October 2021. Sixty-five preclinical studies and 15 clinical trials and open-label studies were selected. Results showed a bias toward studies in depression and, to a lesser extent, schizophrenia. In all disorders, the results were positive in reducing psychiatric deficits. Despite the considerable number of beneficial outcomes reported, the small number of trials and the heterogeneity of protocols make it difficult to draw solid conclusions about the real potency of curcumin in psychiatric disorders.

## 1. Introduction

**Figure 1:**
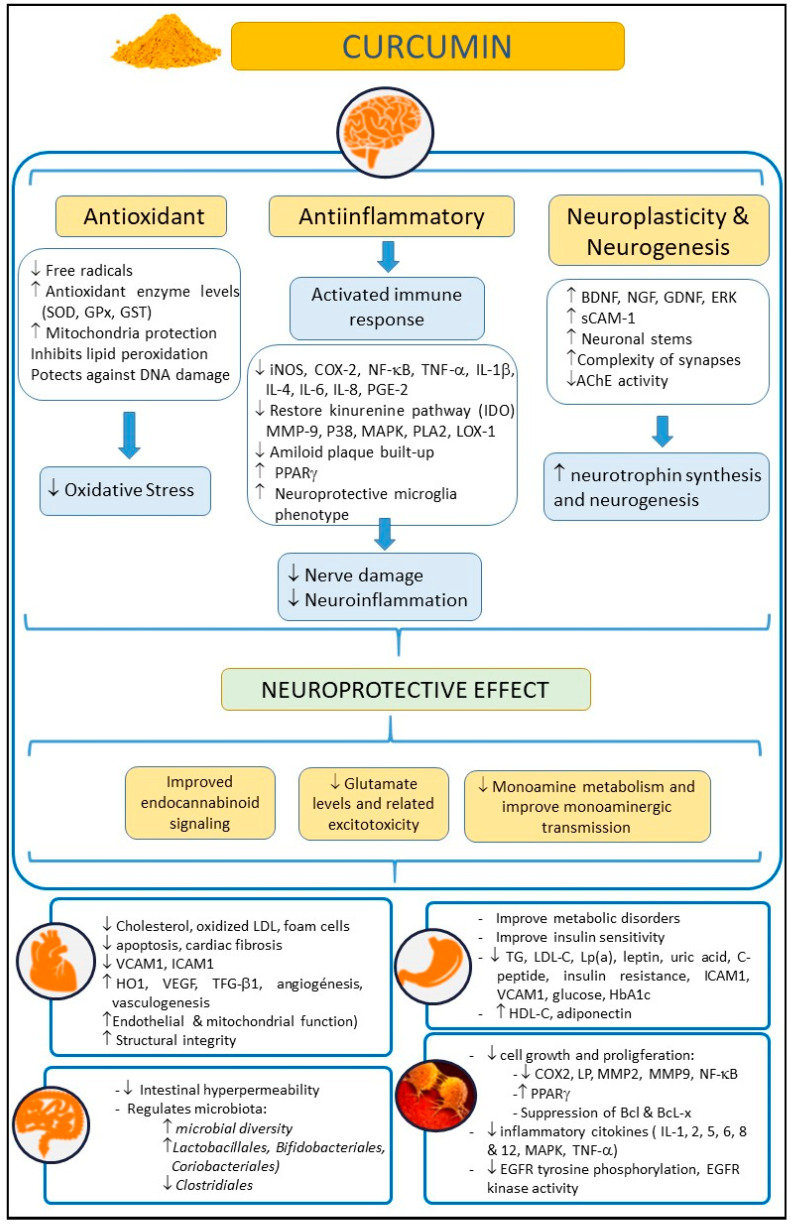
Summary of potential beneficial effects of curcumin on health, in neurological, cardiovascular, intestinal, metabolic, and oncological disorders. Abbreviations: AChE: acetylcholinesterase; Bcl: B-cell lymphoma; BDNF: brain-derived neurotrophic factor; COX-2: cyclooxygenase 2; EGFR: epidermal growth factor receptor; ERK: extracellular-regulated kinase; 9 GDNF: glial cell line-derived neurotrophic factor; GPx: glutathione peroxidase; GSH: glutathione; GST: glutathione S-transferase; HbA1c: glycosylated hemoglobin A1c; HDL-C: high density lipoprotein cholesterol; HO-1: heme-oxygenase-1; ICAM-1: intercellular adhesion molecule-1; IL: interleukin; iNOS: inducible nitric oxide synthase; IL-10: Interleukin-10; LDL-C: low density lipoprotein cholesterol; LOX-1: lectin-like oxidized low-density lipoprotein receptor-1; Lp(a): lipoprotein(a); LP: lipoxygenase; MAPK: mitogen-activated protein kinase; MMP-9: matrix metalloproteinase 9; NF-κB: nuclear factor kappa-B; NGF: Nerve growth factor; PLA2: phospholipase A2; P38: p38 MAKP; PGE-2: prostaglandin E2; PPARγ: peroxisome proliferator-activated receptor gamma; sCAM-1: soluble cell adhesion molecule 1; TG: triglycerides; TNF-α: tumor necrosis factor-α; TGF-β1: transforming growth factor; SOD: superoxide dismutase; VCAM-1: vascular cell adhesion molecule-1; VEGF: vascular endothelial growth factor.

Turmeric (*Curcuma longa*) is an herbaceous plant widely used in Asia as a dye, culinary spice, and as a traditional natural therapeutic compound [1]. The rhizome of this plant, also called turmeric, is enriched with yellow dyes, the curcuminoids [2]. Within this family of compounds, curcumin is considered one of the most relevant. Curcumin, the active compound of turmeric, is a polyphenol that has also been largely used as a remedy for different pathologies in Asia for several decades due to its healthy and biopharmacological properties, and its lack of adverse effects, even at high doses. Moreover, curcumin has been reported to have anti-inflammatory, antioxidant, neuroprotective, and even anti-aging and antineoplasic properties [3,4,5,6,7] (Figure 1). Curcumin may exert its anti-inflammatory and antioxidant (anti-IOS) effects by influencing the synthesis of some IOS regulators, such as heme-oxygenase-1 (HO1), glutathione (GSH), catalase (CAT), and superoxide dismutase (SOD) [8]. These properties cause curcumin to have an impact on those diseases in which IOS regulation does not work correctly and are related to the disease appearance. Thus, curcumin may exert a beneficial effect on the immune system, reducing B lymphocyte proliferation by inhibiting B lymphocyte stimulator (BLYS).

Curcumin can also reduce the neutrophil recruitment to areas affected by inflammation [9], and can also increase the phagocytic activity of macrophages [10]. Furthermore, curcumin has proven to be an effective modulator of the endocrine system, enhancing the uptake or regulating some hormones, such as insulin [11]. All these properties have boosted the interest of researchers in this compound in recent decades. Thus, several preclinical studies and clinical trials have been conducted [12] with the aim of elucidating whether or not curcumin was effective for many different diseases, such as skin [13], cancer, or neurological pathologies [5].

Recently, curcumin has also been used in different psychiatric disorders due to the likely involvement of IOS processes in their onset and evolution. In this sense, the above-described role of curcumin as an anti-IOS drug made this compound a good candidate to halt or palliate the course of these diseases. This is especially important, as current therapeutic strategies for many psychiatric disorders have a relatively high failure rate. Thus, the search for new approaches to help address this problem is ongoing.

So far, several clinical trials and studies with animal models, which we will detail in depth in the following sections of this work, have reported the efficacy of curcumin in some psychiatric disorders, such as depression, schizophrenia, or autism. However, some studies have showed no positive effects of curcumin in neurological diseases. The main and most recommended route of administration of curcumin is oral and, despite considerable high absorption through lipid membranes caused by its lipophilic nature, curcumin has a low bioavailability after being metabolized, accumulating in the spleen, liver, and intestine, with a low uptake in the rest of the organs [8,14,15]. The low absorption by the small intestine and the high metabolism in the liver weaken its oral bioavailability [16], making it necessary to use high oral doses of curcumin to reach other target organs such as the brain [16]. Moreover, its apparent ineffectiveness in interacting specifically with a single pharmacological target has prompted the classification of curcumin as a pan assay interference compound (PAINS) and an invalid metabolic panacea (IMPS) [17,18]. However, despite the poor pharmacokinetics of this compound, the existence of positive results in several studies raises the question of how curcumin could cause a beneficial effect at the brain level despite being barely able to reach this organ. A recent hypothesis explains that curcumin could be acting on the gut microbiota [19] since the intestine and liver are primary sites of metabolism for curcumin [16], reducing intestinal inflammation and, hence, functioning as a neuroprotective agent due to the likely involvement of neuroinflammation in many psychiatric disorders in which alterations of the gut-brain axis play an important role [2,20]. Furthermore, in order to address the low bioavailability of the curcumin, new formulations of this compound are being synthetized to improve its pharmacokinetics and achieve stable curcumin that can reach the brain in a higher concentration. Some of them are based on conjugating curcumin with lipids or co-treating it with piperine, a bio-enhancer that improves the absorption of curcumin [8,21]. A recent emerging and promising strategy to improve its bioavailability in the brain combines curcumin with drug delivery carriers such as liposomes, exosomes, magnetic particles or ultrasound bubbles [22]. Moreover, some of these exosomes have shown an anti-inflammatory capacity [23], which could enhance the anti-inflammatory effect of curcumin in the brain and other organs of interest. Of note, oral and intra-nasal administration of nanoparticles are also being explored, which could increase drug absorption in the brain, representing a great advantage in brain disorders [24,25]. 

Therefore, the aim of this work is to review the current literature on the effect of curcumin and its derivatives in the field of psychiatric disorders, and to discern whether the initial enthusiasm for this compound is well-founded [21,26].

## 2. Materials and Methods

**Figure 2:**
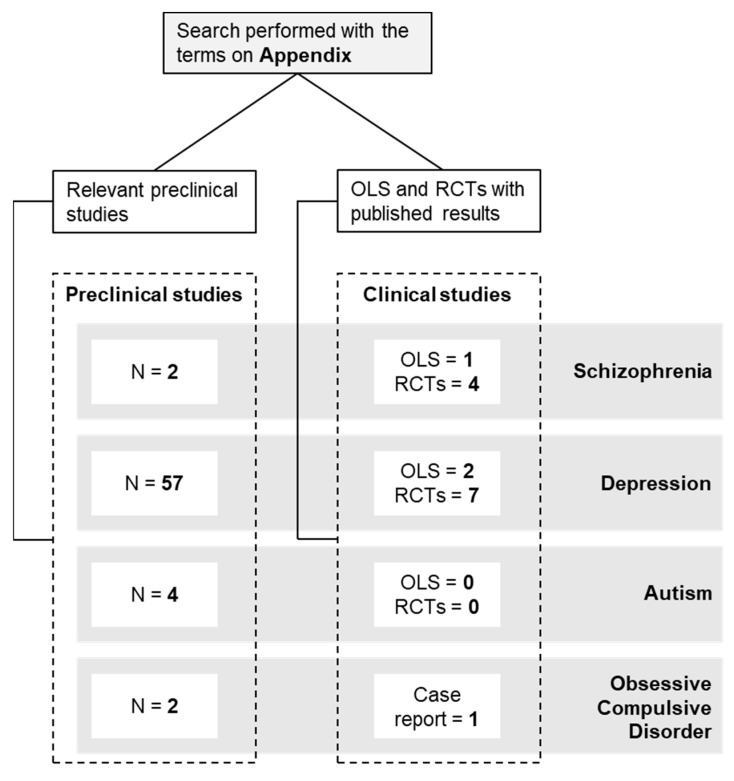
Selected preclinical studies, open-label studies and clinical trials.

A non-systematic literature review was performed in relevant databases until 31st October 2021 (https://ncbi.nlm.nih.gov/pubmed/, https://clinicaltrials.gov/, accessed on 31 October 2021). The data search covered a range from January 2005 to October 2021 (no studies prior 2005 were found). Published English-language studies investigating the effects of curcumin on neuropsychiatric pathologies were included. The search terms used as keywords are listed in the Appendix. All open-label clinical studies (OLS) and randomized clinical trials (RCTs) (Table 1) and preclinical studies (Table 2) that met the search terms were included. For contextualization purposes, one case report was mentioned to explain the rationale for larger trials. Figure 2 shows a scheme with the number of studies for each disorder included in this review. The most commonly used abbreviations can be found in Abbreviation.

## 3. Results

### 3.1. Schizophrenia

Current therapies for schizophrenia mostly focus on treatment with antipsychotics, the prolonged use of which is prone to cause severe extrapyramidal side effects, such as parkinsonism or tardive dyskinesia [27]. In addition, long-term administration of typical antipsychotics decreases antioxidant enzyme levels, thus perhaps participating in the exacerbation of oxidative events [28]. Therefore, the search for new approaches is of great importance. In this regard, the likely involvement of oxidative stress and inflammation in the pathophysiology of schizophrenia has supported the use of curcumin in various preclinical and clinical studies.

On the preclinical side, we only found two studies. In the first one, curcumin-loaded nanoparticles (30 mg/kg, i.p.) were administered to ketamine-treated rats, achieving a reduction in metalloproteases (MMP), adenosine triphosphate (ATP), and mitochondrial enzyme complex II activity in cerebellar mitochondria, along with a reduction in the over-increased locomotor activities in the side-to-side rocking and neck arcing tests [29]. In the second one, published in 2021, the administration of curcumin (30 mg/kg, i.p.) to ketamine-treated mice induced a reduction in oxidative stress biomarkers in the brain, and a reduction in anxiety and depression-like behaviors [30].

On the clinical arena, five studies and trials have been conducted. The first one is an OLS (NCT01875822) in which 17 schizophrenic patients received 1 or 4 g of curcumin or placebo for 16 weeks. However, to our knowledge, there are no published results to date. In 2017, the first randomized, double-blind, placebo-controlled study reporting the effects of curcumin on brain-derived neurotrophic factor (BDNF), a neurotrophin involved in neuroprotection, neuroregeneration and cell survival among other functions, and cognition in 36 patients with schizophrenia and inpatients was published [31]. Patients receiving curcumin (360 mg/day for 8 weeks) showed an increased in BDNF levels relative to baseline and compared to placebo. However, the study failed to find any effect on cognition or other clinical symptoms. In contrast, the three most recent studies showed more promising results as an add-on to antipsychotics in the treatment of negative symptoms (NCT02298985, NCT02476708) or both positive and negative symptoms [32]. The first study, an 8-week randomized, double-blind, placebo-controlled, parallel, fixed-dose pilot clinical trial in 12 patients with schizophrenia, showed that 300 mg of curcumin add-on to conventional medication significantly improved working memory and reduced interleukin-6 (IL-6) levels [33]. The second study, also a randomized, double-blind, placebo-controlled, add-on clinical trial reported an improvement in negative symptoms in 20 patients receiving curcumin (3 g/day, for 24 weeks) compared to 18 patients receiving placebo [34]. Finally, in the third randomized, double-blind, placebo-controlled clinical trial, curcumin (160 mg/day, for 16 weeks) plus usual antipsychotic medication was administered to 28 patients with chronic schizophrenia (28 additional patients received a placebo). Curcumin-treated patients showed an improvement on the negative and positive subscales, the general psychopathology subscale, total Positive and Negative Syndrome Scale (PANSS), Clinical Global Impressions-Severity (CGI-S), and Clinical Global Impressions (CGI-I) scores in comparison with the control group [32].

Therefore, the schizophrenia picture shows an unbalanced proportion of preclinical and clinical studies, biased towards the clinical ones. In all cases, curcumin was well-tolerated and, overall, an improvement of clinical symptoms was observed, especially in negative symptomatology. However, the heterogeneity of doses and curcumin formulations used precludes drawing more robust conclusions.

### 3.2. Depression

Pathophysiology and aetiology of major depression disorder (MDD) are heterogeneous, and traditional antidepressant treatments have some limitations in terms of efficacy, symptom improvement, and side effects. Although the pathological mechanisms are not fully understood, oxidative stress and inflammation seem to play an important role in the pathogenesis of depression, probably through increased inflammatory factors in the central nervous system. In this regard, curcumin has been used and demonstrated to be an effective adjuvant treatment for MDD in several studies.

On the preclinical side, we found a total of 57 studies, 19 of which were performed in mice and 38 in rats. The dose of curcumin ranged from 1 to 300 mg/Kg. The duration of treatment varied from a single intake to a 5-week treatment with curcumin. Regarding the route of administration, 36 used oral administration (23 in the drinking water or food and 13 by gavage), 19 used intraperitoneal administration, and two of them reported no information on the route of administration. In addition, several models of MDD were used, most of them (21) based on a stress-induced model, such as Chronic Unpredictable Mild Stress (CUMS), Single Prolonged Stress (SPS), Chronic Unpredictable Stress (CUS), or Chronic Mild Stress (CMS), while eight of them were induced by surgery (olfactory bulbectomy, ovarectomy, chronic constriction injury or middle cerebral artery occlusion), nine were induced by the administration of reserpine or corticosterone (CORT), and the remaining 19 were induced by other models of MDD.

The antidepressant efficacy of curcumin in modulating depressive behavior in different animal models has been shown in a large number of behavioral studies. Most of the studies reported improved performance in the forced swimming test [35,36,37,38,39,40,41,42,43,44,45,46,47,48], increased locomotor activity in the open field test [49,50,51,52,53,54,55,56,57,58,59,60,61,62,63,64], decreased anxiety in the elevated plus maze test [57,59,64,65,66], improved anhedonia in the sucrose preference test [51,52,54,56,58,62,67,68,69,70,71,72,73,74,75,76], improved short and long-term memory in the passive avoidance test [49,50,55] and water maze test [54,60,77], reduced escape response in the shuttle-box test [78], attenuated the effort-related abnormalities in a choice procedure test [79], and reduced stress in the tail suspension test [50,64,66,69,80,81,82,83,84,85,86,87,88]. Only one study found no improvements in anxiety, as measured by the open field and elevated plus maze tests, nor in “depressive-like” states, as measured by the forced swimming test [89]. Another study found no improvements in anhedonia, as measured by the sucrose preference test [86].

The administration of curcumin has been shown to regulate serotonin (5-HT), dopamine (DA), and noradrenaline (NA) levels. Twenty studies reported an increment of 5-HT levels in the hippocampus, striatum or frontal cortex, which may be due to the interaction found between curcumin and 5-HT/cAMP/PKA/CREB/BDNF-signaling pathway or 5-HT_1A/1B_ and 5-HT_2C_ receptors [35,40,80,83,85,90]. Besides, fifteen studies reported an increased level of DA [38,41,42,44,45,46,48,49,55,72,77,81,87,91]. NA was also incremented in five studies [46,49,55,63,86]. In addition, curcumin has been claimed to present beneficial effects on reducing inflammatory cytokines (IL-1β, IL-6) [60,63,69,70,71,76,77,91], reducing the NF-κB-iNOS-COX-2-TNF-α inflammatory signaling pathway [39,51,52], and modulating the levels of antioxidant markers, such as monoamine oxidase (MAO), malondialdehyde (MDA), CAT, or SOD [43,56,57,62,65,66,82,84,88]. Furthermore, the BDNF is incremented by curcumin treatment [36,39,47,51,54,58,66,67,77,78,90]. Other effects of curcumin have been described in different animal models of depression, such as an interaction with glutamate N-Methyl-D-Aspartate (NMDA) receptors [37], an inhibition of Ca^+2^ channels [74], an increased level of corticosterol and cortisone in plasma [64], or an altered lipid metabolism [75], or an upregulation of the insulin receptor IRS-1 and protein kinase-B (PKB) in the liver [73]. In contrast, only one study reported no effects of curcumin, regarding its interaction with the benzodiazepine site on gamma-aminobutyric acid (GABA) receptor [89].

Only two neuroimaging studies have evaluated the effect of curcumin on brain morphometry and glucose metabolism in an animal model of depression, showing improvements such as a reduction in hippocampal atrophy [50] and an activation of the metabolism of the amygdala in a positron emission tomography (PET) imaging study after curcumin treatment [53].

In the clinical setting, one OLS and eight clinical trials were performed. In 2013, two trials were conducted, one in India (NCT01022632) [92] and one in Israel (NCT01750359) [93]. In the first one, a randomized, active controlled, parallel group trial, curcumin (1000 mg/day) or fluoxetine (20 mg/day) were administered to patients with MDD for 6 weeks (17 patients on fluoxetine alone, 16 patients on curcumin, and 18 patients on fluoxetine/curcumin), which showed no biological effects on depressive symptoms, as measured by the Hamilton Depression Rating Scale (HDRS). In the second study, a randomized, double-blind, placebo-controlled, pilot clinical trial, curcumin (1000 mg/day, for 8 weeks) was administered to 19 patients (27 patients on placebo), showing no improvement in the MDD symptoms measured by the HDRS and the Montgomery–Asberg Depression Rating (MADRS) scales.

In contrast, the remaining trials conducted from 2014 until now showed better results. In 2014, two randomized, double-blind, placebo-controlled trials were conducted in Australia in 25 patients with MDD receiving curcumin (1000 mg/day, for 8 weeks) and 25–27 patients receiving placebo [94,95]. Both studies showed an improvement in MDD symptomatology (IDS-SR30 total score), and the second one also found an increase in some depression-related biomarkers, such as urinary Thromboxane B2 (TBX-B2) and substance-P (SUB-P), and plasma endothelin-1 (ET-1) and leptin levels. Thus, higher levels of these biomarkers were associated with greater reductions in IDS-S30 total scores.

In 2015, one OLS in Iran [96] and a randomized, double-blind, placebo-controlled trial in China were conducted [97]. In the first study, curcumin (1000 mg/day, for 6 weeks) was administered to 61 patients with MDD (50 patients on placebo), showing a decrease in anxiety levels as measured by the Hospital Anxiety and Depression Scale (HADS) and reductions in MDD symptomatology as measured by the Beck Depression Inventory II (BDI-II) scale [96]. Of note, piperine (10 mg/day) was used to increase the bioavailability of curcumin. In the second trial, curcumin (1000 mg/day, for 6 weeks) was administered to 50 patients with MDD (50 patients on placebo), showing an improvement in the HDRS and MADRS scales [97].

In 2017, another randomized, double-blind, placebo-controlled clinical trial was conducted in Australia [98]. The effects of two different doses of curcumin (500 mg/day or 1000 mg/day, for 12 weeks) was evaluated in 28 and 33 patients with MDD, respectively. Both doses induced improvements in symptomatology and anxiety measured by IDS-SR30 and State-Trait Anxiety Inventory (STAI) scales, with no difference between the doses used. In 2018, a randomized, double-blind, placebo controlled trial was performed in 30 patients with MDD treated with an increasing dose of curcumin (500 mg/day to 1500 mg/day with increments of 250 mg/week, for 12 weeks) and 31 on placebo [99]. This escalating medication dosage induced an improvement in the severity of depression on the MADRS scale. Despite this behavioral improvement, no significant effects were found in blood chemistry and electrocardiogram measurements.

Finally, a randomized, placebo-controlled trial is currently in the recruiting phase (NCT04744545 2021). The study estimates to recruit 60 patients with MDD, with curcumin (1500 mg/day) as an adjuvant treatment for MDD.

### 3.3. Autism Spectrum Disorder (ASD)

Although the etiology of this disorder is largely unknown, oxidative stress and inflammation have been hypothesized to be key factors in its occurrence, especially through an exacerbated increase in pro-inflammatory metalloproteases. In this sense, the anti-inflammatory and antioxidant potential of curcumin could be effective in alleviating this disorder.

So far, no clinical trials have been conducted in patients with ASD. On the preclinical field, only four studies have been performed in animal models, two in rats and two in mice. The first study used a model based on the intracerebroventricular injection of propanoic acid (PPA) in Sprague-Dawley rats. After the PPA injection, curcumin was orally administered for 4 weeks at different doses (50/100/200 mg/kg). The treatment restored many behavioral defects in PPA rats, such as social interaction, anxiety, depression, and repetitive behaviors. In addition, curcumin reduced the levels of MMP-9 and Thiobarbituric Acid Reactive Substances (TBARs), increased the activity of GSH, CAT, and SOD, and restored normal function of mitochondrial enzyme complex 1 [100]. In 2017, another study, based on prenatal valproic acid (VPA) exposure to fetal Wistar rats, proposed early postnatal administration of curcumin (first seven days after birth). This approach was reported to restore oxidative stress deficits and the abnormal body and brain weight values [101]. Two subsequent studies were performed in the BTBRT^+^ltpr3^tf^/J (BTBRT) mouse model. The first one, in which curcumin (20 mg/kg) was administered from PND 6 to 8, reported enhanced neural stem cell proliferation, along with increased sociability and improved short-term memory [102]. The second study evaluated three different doses of curcumin (25/50/100 mg/kg), showing restoration of different oxidative stress markers in the hippocampus and cerebellum, along with a dose-dependent increase in sociability in curcumin-treated mice [103].

Taken together, these results suggest that curcumin could be effective in preventing some autistic behavioral and biochemical traits, but the lack of clinical trials do not allow for drawing solid conclusions.

### 3.4. Obsessive Compulsive Disorder (OCD)

The etiology of OCD is not fully understood either, but it has been hypothesized that it is a result of the existence of a deficit of monoamines in specific brain regions such as the orbitofrontal cortex and the anterior cingulate gyrus. In this regard, the potential of curcumin as an inhibitor of MAO-A and MAO-B, both of which are involved in monoamines degradation [97], led researchers to test its efficacy as an adjuvant treatment in this disorder.

Only two preclinical studies have been conducted to date. The first, carried out in 2010 by Jithendra and Murthy, evaluated the potential of orally administered curcumin (5 or 10 mg/kg) as a therapeutic approach to reduce obsessive-compulsive signs in the quinpirole-induced OCD rat model. Following treatment with both doses, a reduction in brain DA levels, together with an increase in serotonin levels, was observed in curcumin-treated pathological rats. In addition, an improvement in obsessive-compulsive symptoms together with a protective effect on the water maze memory task at both doses was reported [97]. The second study was recently conducted, in 2021, by Mishra et al. In this work, they intraperitoneally administered ethanolic extract of curcumin (10, 15, 25, or 40 mg/kg) to Swiss albino mice that had poor performance in the marble-burying behavior (MBB) and motor activity (MA) tests. The treatment at the dose of 40 mg/kg resulted in improved performance in the MBB test, but not in the MA [98].

From a clinical point of view, no OLS or trials have been conducted to date. However, a case report was announced in 2018. In this case, a 3-year-old child with a diagnosis of OCD and tics was treated with a combination of N-acetylcysteine (dose increase from 600 to 1800 mg/day) and curcumin (90 mg/day). After 7 days, a complete remission of tics and OCD symptoms was observed. Finally, after 3 weeks, symptoms remitted completely, together with a drastic reduction in Children’s Yale–Brown Obsessive Compulsive (CY-BOCS) and Yale Global Tic Severity (YGTSS) total scores [99].

Taken together, these data do not shed enough light to conclude whether curcumin is an effective compound for the treatment of OCD, especially in the case report, in which the observed positive effect could also be attributed to the administration of NAC.

## 4. Discussion

Anti-inflammatory, antioxidant, and neuroprotective properties of curcumin, along with many multi-target beneficial effects, such as the modulation of monoamine synthesis, have exponentially promoted the investigation of its properties during this last decade. Two-hundred and ninty-six articles containing research on curcumin were published in the PubMed database in 2005. In 2010, this number increased to 714 and, in 2020, to 2130. The field of psychiatry has not been immune to this boost. The likely involvement of oxidative stress, inflammation, and monoamine deficits in the pathophysiology of many psychiatric disorders, together with the poor response to current therapies in a significant proportion of these patients, have pushed researchers to investigate new therapeutic compounds that could improve current treatments. In this work, we reviewed the literature on the effects and efficacy of curcumin and its derivatives in four psychiatric disorders: schizophrenia, depression, autism, and obsessive-compulsive disorder (OCD). A total of 65 preclinical studies and 14 clinical trials were reported. Most of these studies were conducted on depression, approximately 88% were preclinical studies and 64% were clinical studies. In all disorders, curcumin was well tolerated, with no harmful side effects. This was not surprising, as curcumin has been used for the last centuries as an additive spice in East Asian cuisine. Moreover, curcumin was shown to be beneficial in palliating or reversing symptoms associated with psychiatry in all the studies analyzed and completed, with the exception of one preclinical and two clinical studies in depression, which reported no improvement [89,92].

As mentioned above, the percentage of studies on depression, as compared to autism and OCD, is highly unbalanced. This fact (no clinical trial on the effect of curcumin on either autism or OCD has been conducted so far) prevents us from drawing solid conclusions on the possible effectiveness of curcumin in these disorders. This large bias towards studies on depression could be explained by a likely predisposition of patients with depression to use new therapies compared to psychotic or autistic patients. Nevertheless, we believe that the efforts directed to the synthesis of new formulations of this compound, together with an improvement of its pharmacokinetic properties, will increase the interest in curcumin and decrease the reluctance to use it in more psychiatric disorders, such as OCD or autism.

In the case of schizophrenia, the reported outcomes showed a beneficial effect of curcumin in both preclinical and clinical studies. In clinical trials, curcumin proved to be effective in alleviating both positive and negative symptoms of schizophrenia when administered together with regular antipsychotic medication. The clinical relevance of these results could be of great importance, due to the adverse events that can be caused by the extensive and chronic use of antipsychotics. Besides, its excessive use can lead to a paradoxical increase in oxidative stress and inflammation. In addition, some widely used antipsychotics, such as clozapine, are able to activate hepatic sterol regulatory element-binding proteins (SREBPs) and enhance downstream lipogenesis, leading to an increase in lipid peroxides and brain phospholipase A2 (PLA_2_), which can lead to cell death [104]. In this sense, curcumin could exert its beneficial effect in schizophrenia through an inhibition of PLA_2_ enzyme [105]. Nevertheless, the heterogeneity of the protocols used in these studies, in terms of curcumin doses and stage of the disorder, makes it difficult to make comparisons between trials and draw a solid conclusion.

In depression, we found the vast majority of studies, in both preclinical and clinical domains, showed some beneficial effect of curcumin in reducing symptoms associated with depression. In addition to the recognized role of curcumin as an anti-inflammatory and antioxidant agent, positive improvement of depressive deficits could be exerted through modulation of the indolamine 2,3-dioxygenase (IDO) enzyme, involved in the kynurenine pathways and, thus, in the inhibition of serotonin synthesis. Curcumin treatment was shown to be able to counteract the action of this enzyme [76,106]. Therefore, the overall effect of curcumin in this disorder seems to be mainly positive.

Even though the results we have found and show here are overwhelmingly positive, there is a significant amount of literature warning about this compound, especially concerned about its poor pharmacokinetics and chemical instability, and its non-specific multi-target effects [17,18]. Although the results presented in this review pointed in a different direction, we considered it relevant to mention, at least, these discordant voices which claim that curcumin is an unstable compound with barely therapeutic efficacy.

Finally, even though this review provides a thorough review of the current literature, there are several limitations. First and foremost, the great heterogeneity of methodologies used in all the studies has hindered the possibility of making comparisons between studies. This has been especially relevant in the case of the different formulations of curcumin and the doses used. Secondly, the small number of trials and clinical studies carried out in some of the pathologies mentioned, together with the small number of participants in some of them, prevents us from drawing solid conclusions. Thirdly, the small number of trials in some cases forced us to compare trials of the same disease but focused on different stages of the disease or on adjuvant treatments. Although this work was conducted after an exhaustive search in well-known databases, there is always an intrinsic limitation derived from the non-systematic nature of this review. One final remark derives from the well-known problem of publication bias towards positive results, which may prevent some negative-result studies from being reported in high impact journals, or even published at all.

## 5. Conclusions

Overall, curcumin, due to its anti-inflammatory and antioxidant properties, has been shown to be effective in the vast majority of the studies presented. However, the lack of homogeneity of the protocols used and the scarce number of trials prevents us from concluding whether curcumin is really a useful therapeutic tool in the psychiatric field.

## Figures and Tables

**Table 1:** Clinical studies and trials in neuropsychiatric diseases.

Disease	Authors	Type	Phase	N Patients	Type Patients	Country	Duration	Dosage	Other Treatment	Biological Effects	Clinical Efficacy	Safety and Tolerability
Schizophrenia	NCT01875822	Open-label study	1–2	17	Schizophrenia patients	Puerto Rico	16 weeks	1000 or 4000 mg CUR	5 mg Bioperine + Antipsychotic	-	-	-
	Wynn and Green, 2017 (NCT02104752)	Randomized, double-blind, placebo-controlled study	1–2	36 (17 CUR, 19 placebo)	Schizophrenia patients and inpatients	United States	8 weeks	360 mg/day of Theracurcumin	-	Increase in plasma levels of BDNF in CUR patients	No effect on clinical symptoms	No significant adverse events
	Kucukgoncu et al., 2019 (NCT02476708)	Randomized, double-blind, placebo-controlled, add-on study	-	12 (6 CUR, 6 placebo)	Schizophrenia outpatients	United States	8 weeks	180 mg/day of Theracurcumin	Usual antipsychotic medication	Reduction in IL-6 in CUR SCZ patients	Significant improvement in working memory. No significant improvement in cognitive domains, negative symptoms and total PANSS	No significant adverse events
	Miodownik et al., 2019 (NCT02298985)	Randomized, double-blind, placebo-controlled, add-on	4	38 (20 CUR, 18 placebo)	Schizophrenia outpatients	Israel	24 weeks	3000 mg/day	Usual antipsychotic medication	-	Improvement in total PANSS and in the negative symptoms subscale. No changes in positive and general PANSS subscales nor the CDSS	No significant adverse events
	Hosseininasab et al., 2021	Randomized, double-blind, placebo-controlled, add-on trial	-	56 (28 CUR, 28 placebo)	Chronic and stable schizophrenia inpatients	Iran	16 weeks	160 mg/day	Usual antipsychotic medication	-	Improvement in total PANSS and in the negative symptoms subscale, general psychopathology subscale, positive subscale, total PNSS, CGI-S, and CGI-I. No changes in Extrapiramidal symptom rating scales nor CDSS	No significant adverse events
Depression	Sanmukhani (2013) NCT01022632	Randomized control trial	-	51 (17 fluoxentine, 16 CUR, 18 fluoxentine + CUR)	MDD patients (>18 years)	India	6 weeks	1000 mg/day of CUR; 20 mg/day of Fluoxentine	Paracetamol and diazepam	Not described	No significant effects produced by curcumin or its combination with fluoxentine (HDRS)	Curcumine was well tolerated
	Bergman (2013) NCT01750359	Randomized, double-blind, placebo-controlled, pilot clinical trial	4	39 (19 CUR, 20 plecebo)	MDD patients (20–81 years)	Israel	5 weeks	500 mg/day	Escitalopram and venlafaxine XR	Not described	No effects of curcumin (MADRS and HDRS)	No adverse effects during the treatment
	Lopresti (2014)	Randomized, double-blind, placebo-controlled trial	-	25 (curcumine 1000 mg/day), 27 (placebo)	MDD patients (20–65 years)	Australia	8 weeks	1000 mg/day	SSRIs and SNRIs	Not described	Long-term improvement in IDS-SR30 total scores. Long-term improvement in STAI anxiety scores	Minor severity side effects
	Lopresti (2014)	Randomized, double-blind, placebo-controlled trial	-	25 (curcumine 1000 mg/day), 25 (placebo)	MDD patients (20–65 years)	Australia	8 weeks	1000 mg/day	Non-specified antidepressant medication	Increased urinary TBX-B2 and SUB-P. Higher plasma ET-1 and leptin levels	Improvement in IDS-SR30 total score	Minor severity side effects
	Panahi (2015)	Open-label study	-	111 (61 CUR + piperine, 50 placebo)	MDD patients (18–65 years)	Iran	6 weeks	1000 mg/day of CUR + 10 mg/day of piperine	TCAs, BZDs, SSRIs and SNRIs	Not described	Improvements in HADS total score. Reductions in BDI-II total score	Not described
	Yu (2015)	Randomized, double-blind, placebo-controlled pilot study	-	100 (50 CUR, 50 placebo)	MDD patients (31–59 years)	China	6 weeks	500 or 1000 mg/day of CUR; 30 mg/day of Saffron	Escitalopram	Decrease in IL-1β, TNF-α and salive cortisol concentrations. Increase in NF levels in plasma	Improvement in HDRS and MADR total scores	No adverse effects
	Lopresti and Drummond (2017)	Randomized, double-blind, placebo-controlled study	-	123 (28 CUR, 33 CURX2, 26 CUR +Saffron, 36 placebo)	MDD patients (18–65)	Australia	12 weeks	1000 mg/day or 500 mg/day	Non-specified antidepressant medication	Not described	Improvements in IDS-SR30 and STAI total scores after the combination of treatments. No differences between different doses of curcumin	Minor severity side effects
	Kanchanatawan (2018)	Randomized, double-blind, placebo-controlled study	-	61 (30 CUR, 31 placebo)	MDD patients (18–63)	Thailand	12 weeks	500 mg/day to 1500 mg/day with an increment of 250 mg/week	Fluoxentine, SSRis, mianserin, trazodone, sodium valproate, propanolol and enalapril	No significant effects on blood chemistry and ECG measurements	Improvement in MADRS total score.	No adverse effects
	NCT04744545 (2021)	Randomized, placebo-controlled trial	-	60 (estimated)	MDD patients (>18 years)	Canada	12 weeks	1500 mg/Kg	Integrative treatment program based on several evidence-based practices and overseen by licensed clinical therapists that is delivered via a Smartphone app	-	-	-
Obsessive Compulsive Disorder	Moore and Nat (2018)	Case report	-	1	One case report	United States	3 weeks	90 mg/day og CUR; 600–1800 mg/day of NAC	Not specified	Not described	Reduction in CY-BOCS and YGTSS total scores	Not described

**Table 2:** Preclinical studies in neuropsychiatric diseases.

Pathology	Reference	N Animals	Animal Model	Treatment	Biological Effects	Behavioral Effects
Schizophrenia	Naserzadeh et al., (2018)	24 (6/group)	Ketamine-treated Wistar male rats	CUR-loaded magnetic nanoparticles (17 mg/300 ul PBS), i.v.	Reduction in MMP, ATP and mitochondrial complex II activity in mitochondria of the cerebellum	Reduction in the over-increased locomotor activities (side-to-side rocking and arcing of neck) in the CUR-treated ketamine rats, without reaching the control values.
	Moghaddam et al., (2021)	35 (7/group)	Ketamine-treated, during the last 15 days of CUR administration mice	Curcumin-loaded nanophytosomes (20 mg/kg) during 30 days	Reduction in biomarkers of oxidative stress in cortical and subcortical regions	Reduction in anxiety in CUR-treated ketamine mice. Reduction in depressive-like behaviors in ketamine mice treated with CUR
	Xu et al., 2005	60 (6/group)	Regular male ICR mice	1.25, 2.5, 5, or 10 mg/Kg 30 min before tests, p.o.	Increment of 5-HT and DA in the frontal cortex and striatum at high doses. Inhibition of monoamine oxidase activity	High doses improved the forced swimming and tail suspension tests. No effect on locomotor activities
	Xu et al., 2005	72–84 (6–7/group)	OB male SP rats	1.25, 2.5, 5, or 10 mg/Kg, 14 days, p.o.	Reversion of the deficits of 5-HT and NA in hippocampus and frontal cortex; 5-HIAA and DOPAC in hippocampus and DA in frontal cortex	Improvement in forced swimming, open field, and passive avoidance tests
	Xu et al., 2006	36 (6/group)	Chronically stressed male SP rats	2.5, 5, or 10 mg/Kg, 21 days, p.o.	Reversed the effects on adrenal gland size and weight. Blocked the stress-induced decreases in BDNF and pCREB/CREB	High doses improved the effects in shuttle-box test
	Xu et al., 2007	30–35 (5–6/group)	Chronically stressed male SP rats	5, 10, or 20 mg/Kg, 21 days, p.o.	Improved hippocampal neurogenesis and blocked the decrease in 5-HT1A mRNA and BDNF protein levels in the hippocampal subfields	-
Depression	Kulkarni et al., 2008	30 (6/group)	Reserpine treated male Laca rats	10–80 mg/Kg, 60 min before tests, i.p.	Increment of 5-HT, DA levels, MAO-A and MAO-B at higher doses	Dose dependent improvement in forced swimming test
	Wang et al., 2008	40–48 (10–12/group)	PCPA male ICR mice	2.5, 5, or 10 mg/Kg, 45 min before tests, p.o.	Interaction with 5-HT_1A/1B_ and 5-HT_2C_ receptors	Improvement in forced swimming test
	Li et al., 2009	56 (7/group)	CUMS male Wistar rats	15 or 30 mg/Kg, 4 weeks, i.g.	Reduced serum corticosterone levels. Enhanced AC activity and cAMP levels and upregulated several AC subtypes in the hippocampus, cortex, and hypothalamus. Increased 5-HT levels	Improvement in sucrose preference test
	Bhutani et al., 2009	36 (6/group)	Chronically stressed female Wistar rats	20 or 40 mg/Kg, 21 days, i.p.	Dose dependent reduction in MAO-A and MAO-B. Reversed the effects on NE, DA, and 5-HT levels	Dose dependent improvement in forced swimming test
	Arora et al., 2011	48 (8/group)	Reserpine treated male Wistar rats	100, 200, or 300 mg/Kg, 2 days, i.p.	Dose dependent reversion of NE, DA and 5-HT reduced levels. Increment of SUB-P concentration, nitrodative stress, inflammatory cytokines, NF-κβ and caspase-3 levels in hippocampus and cortex	Reduced the deficits in Randall Sellitto and von-Frey hair tests. Improvement in forced swimming test
	Huang et al., 2011	18 (6/group)	CORT-treated male SP rats	20 mg/Kg, 21 days, p.o.	Increment of BDNF levels induced by CORT treatment in hippocampus and frontal cortex	Improvement in forced swimming and sucrose preference tests
	Kulkarni et al., 2011	Not specified	Regular male Laca mice	50–200 mg/Kg, 30 min before tests, p.o.	Increment of 5-HT at low doses and DA at high doses	Dose dependent improvement in forced swimming test
	Zhang et al., 2012	60–75 (10–12/group)	SL327 male C57BL/6 mice	40 mg/Kg, 21 days, i.p.	Improvement of ERK deregulation on BDNF expression in the amygdala	Improvement in forced swimming test
	Borre et al., 2013	40–48 (10–12/group)	OB or ZnSO4 anosmia-induced male SP rats	20 g/day of 0.25 mg/Kg curcumine diet, 42 days	Reduced hippocampal atrophy and decreased the peripheral immune activation	Attenuation of cognitive and behavioral deficits in open field, tail suspension, passive avoidance, T-maze and holeboard tests
	Rinwa et al., 2013	50 (5/group)	OB male Wistar rats	100, 200, or 400 mg/Kg, 2 weeks, p.o.	Dose dependent reversion of TNF-α, caspase-3 and BDNF levels	Dose dependent improvement of forced swimming, sucrose preference and open field tests
	Lin et al., 2013	40 (6–14/group)	CUS male SP rats	40 mg/Kg, 30 days, p.o.	Strong deactivation of the left primary auditory cortex and activation of the amygdalohippocampal cortex	Improvement in sucrose preference and open field tests
	Hurley et al., 2013	32 (8/group)	Male Wistar Kyoto rats	50, 100, or 200 mg/Kg, 10 days, i.p.	Dose dependent increase in hippocampal BDNF levels	Improvement in forced swimming test but no effects on open field test
	Jiang et al., 2013	40 (10/group)	CMS male Wistar rats	10 mg/Kg, 3 weeks, i.g.	Inhibited cytokine gene expression at mRNA and protein level and reduced the activation of NF-κβ	Reduced sucrose preference and decreased locomotor activity in open field test
	Zhang et al., 2013	40–48 (10–12/group)	NMDA receptor antagonists treated male Kun-Ming mice	10, 20, or 40 mg/Kg, 45 min before tests, i.p.	Interaction with glutamate-NMDA-receptors	Improvement in forced swimming test
	Zhao et al., 2013	24–36 (8–12/group)	CCI in male ICR mice	5, 15, or 45 mg/Kg, 3 weeks, p.o.	Interaction with 5-HT_1A_ and GABA receptors	Dose dependent improvement in forced swimming and tail suspension tests
	Wang et al., 2014	40(10/group)	LPS treated male Kun-Ming mice	50 mg/Kg, 7 days, i.p.	Attenuated LPS induced microglial activation and over production of pro-inflammatory cytokines, levels of inductible nitric oxide synthase and cyclooxygenase-2 mRNA in the hippocampus and prefrontal cortex	Improvement in forced swimming, tail suspension, and sucrose preference tests
	Liu et al., 2014	40 (10/group)	CUS male Wistar rats	10 mg/Kg, 5 weeks, i.g.	Increased hippocampal BDNF and ERK levels	Reduced sucrose preference and impaired learning and memory function in open field and Morris water maze tests
	Cui et al., 2014	48 (8/group)	CUMS male SP rats	10, 40, or 80 mg/Kg, 30 min before tests, i.g.	Improved the activity of anti-oxidant enzymes and energy metabolism enzymes	Improvement in open field and sucrose preference tests
	Zhang et al., 2014	64 (16/group)	CUMS male Wistar rats	40 mg/Kg, 6 weeks, i.p.	Reverted the effects on the expression of BDNF, PSD-95 and synaptophysin in the lateral amygdala	Improvement in open field, forced swimming and sucrose preference tests
	Haider et al., 2015	24 (6/group)	Stressed male Wistar rats	200 mg/Kg, 1 week, p.o.	Improved the levels of MDA, CAT, GPx, SOD and AChE	Improvement in elevated plus maze, open field and forced swimming tests
	He et al., 2016	Not specified	CORT-treated female C57BL/6 mice	20 mg/Kg, 2 weeks, i.p.	Improvement of DA levels in blood. Increase in neurotransmitters in hippocampus and striatum. Increased expression of CBR1, p-MEK1, and p-ERK1/2	Improvement in forced swimming and rotarod tests
	Chang et al., 2016	30 (6/group)	OB male Wistar rats	10, 20, or 40 mg/Kg, 45 days, p.o.	Reversed the effects on NA, 5-HT, 3, DOPAC acid and 5-HIAA in the hippocampus. Normalized the levels of DA, NA and 5-hydroxyindoleaceticacid in the prefrontal cortex	Improvement in passive avoidance and open field tests
	Yusuf et al., 2016	42 (6/group)	Stressed albino mice	2.5, 5, 10, or 20 mg/Kg, 60 min before tests, i.p.	Increase in SOD catalase activity	Improvement in force despair, forced swimming and tail suspension tests
	Demir et al., 2016	34(7–10/group)	Cisplatin treated male Wistar rats	300 mg/Kg, 5 weeks, p.o.	-	Improvement in forced swimming, open field and elevated plus maze tests
	Shen et al., 2017	48 (6/group)	CMS male SP rats	15, 30, or 60 mg/Kg, 33 days, p.o.	Upregulation of IRS-1, Akt in the liver and reversed metabolic abnormalities	Improvement in glucose preference test
	Yohn et al., 2017	45 (9/group)	Tetrabenzamine treated male SP rats	80–160 mg/Kg, p.o. or 2–8 ul/Kg infusions into ventricles	-	Attenuated the effort-related abnormalities in a choice procedure test
	He et al., 2017	Not specified	CORT administration in C57BL/6 mice	20 mg/Kg, 3 weeks	Increased DA/5-HT levels, CB1 mRNA levels and CB1, p-MEK1, and p-ERK1/2 protein expression levels in the hippocampus and striatum. Increment on CBR1 expression and proliferation of astrocytes in the hippocampus and striatum	Improvement in forced swimming test
	Choi et al., 2017	16 (4/group)	Chronically stressed male SP rats	50 or 100 mg/Kg, 18 days, p.o.	Rescued the attenuated BDNF expression and inhibited the enhancement of COX-2 expression	Improvement in forced swimming test
	Ceremuga et al., 2017	55 (11/group)	Flumazenil treated male SP rats	20 mg/Kg, 10 min before tests, i.p.	No interaction between curcumin and benzodiazepine site of the GABAs receptor was observed	No effects on forced swimming, open field and elevated plus maze tests
	Vasileva et al., 2018	48 (6–7/group)	CMS-LPS treated male Wistar rats	20 mg/Kg, 8 days, i.g.	Reversion of the increase in cytokine levels	Improvement in open field and water maze tests
	Lee and Lee, 2018	42–49 (6–7/group)	SPS male SP rats	20, 50, or 100 mg/Kg, 14 days, i.p.	Recover of neurochemical abnormalities and decreases of 5-HT in the hippocampus, amygdala, and striatum	Improvement in elevated pluz maze, fear conditioning and open field tests
	Fan et al., 2018	24 (8/group)	CUMS male Wistar rats	40 mg/Kg, 5 weeks, i.p.	Repression of the inflammatory response and neuronal structural abnormalities produced by CUMS	Improvement in forced swimming and sucrose preference tests
	Lian et al., 2018	36 (6/group)	Regular male ICR mice	2, 5, or 10 mg/Kg, 1–24 hs before tests, i.g.	Activation of 5-HT_1A_/cAMP/PKA/CREB/BDNF-signaling pathway	Improvement in forced swimming and tail suspension tests. No alteration in open field test
	Fidelis et al., 2018	35–40 (7–8/group)	β-amyloid treated Swiss male mice	10 mg/mL, 12 days, i.g.	Reduced Aβ-oxidative stress via SOD and CAT in the prefrontal cortex	Improvement in forced swimming and tail suspension tests. No changes in open field test
	Abd-Rabo et al., 2019	70 (14/group)	Ovariectomized female Wistar rats	100 mg/Kg, 30 days, p.o.	Improvement of serotonin content by upregulating 5-HT_1A_ and down regulating monoamine oxidase	Improvement of forced swimming test
	Fan et al., 2019	72 (18/group)	CUMS male Wistar rats	40 mg/Kg, 5 weeks, i.p.	Reduced the expression of IL-1β and inhibited neuronal apoptosis within neurons of the ventromedial prefrontal cortex	Improvement in forced swimming and sucrose preference tests
	Mohammed et al., 2019	65 (11–15/group	Reserpine treated male Wistar rats	20 mg/Kg, 7 or 15 days, i.p.	Restored DA and 5-HT levels, but not NE levels after 7 days of treatment. Increase in alpha and beta 2-waves, tetha and beta 1, and decrease in delta waves	Improvement in forced swimming test
	Madiha and Haider, 2019	30 (6/group)	Rotenone treated Wistar rats	100 mg/Kg, 2 weeks, p.o., pre- and post-Rotenone	Reverted DA and 5-HT levels in striatum and hippocampus	Improvement in social interaction and sucrose preference test
	Zhang et al., 2019	18–21 (6–7/group)	CUMS male SP rats	100 mg/Kg, 4 weeks, i.g.	Reduced the expression of IL-1β, IL-6, and TNF-α and suppressed activation of NF-κβ. Inhibited the P2 × 7R/NLRP3 inflammasome axis activation, and reduced the synthesis of IL-1β. Ammeliorated the activation of IDO and increased kynurenine/tryptophan ratio	Improvement in forced swimming, elevated pluz maze and sucrose preference tests
	Liao et al., 2020	24 (8/group)	CUMS male SP rats	100 mg/Kg, 4 weeks, i.g.	Decrease in protein expression of stress markers and increase in CAT. Reversed the inhibition of Nrf2-ARE signaling pathway and increased mRNA expression of NQO-1 and HO-1. Increased the ratio ofpCREB/CREB and BDNF, PSD-95 and synaptophysin	Improvement in forced swimming, open field, novelty-suppressed feeding, and sucrose preference tests
	Qi et al., 2020	35 (6/group)	Reserpine treated male ICR mice	5 mg/Kg, i.g. or 14.6, 29.2, 58.4 ug/Kg, nasal, 1 h before tests	Increase in NE, DA, 5-HT and their metabolites in hippocampus and striatum	Improvement in forced swimming and tail suspension tests
	Wang et al., 2020	18 (6/group)	MCAO and CMS male SP rats	100 mg/Kg, 4 weeks, i.g.	Blocked Ca^+2^ accumulation, inhibited the activation Ca^+2^ channels	Improvement in forced swimming and sucrose preference tests
	Li et al., 2020	50(10/group)	Regular male ICR mice	1, 3, or 9 mg/Kg, 3 days, i.g.	Modulated 5-HT_1A_-dependent cAMP/PKA/pCREB/BDNF signaling pathway	Improvement in forced swimming and tail suspension tests in a dose dependent manner
	Abu-Taweel and Al-Fifi, 2020	60 (6/group)	Mercury chloride treated male Swiss mice	150 or 300 ppm, 36 days, p.o.	Dose dependent improvements of corticosterol and cortisone levels in plasma	Dose dependent improvements in forced swimming, tail suspension, open field and plus maze tests
	He et al., 2020	24 (3/group)	CORT treated CBR1+/+ and CBR1-/- mice	20 mg/Kg, 2 weeks, i.p.	Increased mRNA and protein expression levels of neuronal markers, MEK and Tuj1. Increase in released DA and NE and the mRNA expression of CBR1 and the downstream of genes Rasgef1c and Egr1	Improvement in forced swimming test
	Zhang et al., 2020	24–27 (8–9/group)	TN male SD rats	45 mg/Kg, 27 alternative days, i.g.	Altered ether lipid metabolism and glycerophospholipid metabolism	Improvement in forced swimming and sucrose preference tests
	Da Silva-Marques et al., 2020	40–52 (10–13/group)	CUMS male Swiss mice	50 mg/Kg, 28 days, p.o.	Increase in CAT levels in the brain. No potential renal and hepatic damage	Improvement in forced swimming and elevated plus maze tests
	Saied et al., 2021	50 (7–10/group)	OVX female albino rats	100 mg/Kg, 30 days, p.o.	Modulated DA and NE levels, downregulated MAO-B and upregulated tyrosine hydroxylase and DA receptors in the limbic region. Reduced the production of corticosterone, IL-1β, IL-6, and nitric oxide. Normalized the levels of MDA	Improvement in the open field test
	Afzal et al., 2021	24 (8/group)	CRS male Wistar rats	200 mg/Kg, 1 week	Reverted the effects on hippocampal BDNF, 5-HT, DA, and Ach levels	Improvement in Morris water maze and pattern separation tests
	Rubab et al., 2021	40 (5/group)	LPS administration in male SP rats	40 mg/Kg, 8 days, i.p.	Suppressed the expression of BDNF, TNF-α, p-NF-κβ, and COX-2	Improvement in forced swimming, tail suspension, elevated plus maze, and light-dark box tests
	Pan et al., 2021	45 (9/group)	Regular ICR male mice	10 mg/Kg, 3 days, p.o.	Increased levels of 5-HT and NA in the hippocampus and frontal cortex. Inhibition of MAO-A activity	Improvement in forced swimming, tail suspension tests. No effects on sucrose preference and novelty suppressed feeding tests
	Khadrawy et al., 2021	21 (7/group)	Reserpine treated male Wistar rats	5 mg/Kg, 14 days, i.p.	Reversion of the levels of MAO, AchE, Na^+^, K^+^, and ATPase	Improvement in forced swimming test
Autism	Bhandari and Kuhad, 2015	40 (5/group)	Intracerebroventricular injection of PPA in male SP rats	50, 100, or 200 mg/kg/day, during 4 weeks, p.o.	Reduction in the (TBARS) in CUR animals. Increase in glutathione, superoxide dismutase and catalase levels in CUR rats’ brains. Restoration of mitochondrial enzyme complex I activities in CUR rats. Dose-dependent reduction in MMP-9 in PPA rats treated with CUR	Dose-dependent improvements of social skills in CUR-treated PPA animal. Improvement in locomotor activity, rotarod, elevated plus maze and open field tests, especially at 200 mg/kg/day.
	Al-Askar et al., 2017	40(10/group)	Fetal exposition (GD12.5) to VPA in Wistar rats	1 mL, oral, for 7 days after birth	Increase in brain and body weight in CUR-treated VPA animals. Depletion of IFN-γ in VPA rats with curcumin treatment. Partial restoration of IL-6 and glutamate normal levels in VPA rats with CUR	-
	Zhong et al., 2020	48 (12/group)	BTBRT+ltpr3tf/J mice	20 mg/kg, from PND 6 to PND 8, i.p.	Enhancement of neural stem cell proliferation in BTBRT mice treated with CUR	Improvement in 3-chambered social approach and novel object recognition tests. No effect of CUR in male–female reciprocal social interaction. No changes in anxiety nor locomotor activity caused by CUR
	Jayaprakash et al., 2021	54 (7/group)	BTBRT+ltpr3tf/J mice	25, 50, or 100 mg/kg, 1 week before tests, i.p.	Restoration of catalase and superoxide dismutase in hippocampus and cerebellum of CUR-treated BTBRT mice	Dose-dependent increase in sociability in CUR-treated mice
Obsessive Compulsive Disorder	Jithendra and Murthy, 2010	30 (6/group)	Quinpirol treated Wistar rats	5 or 10 mg/Kg, 35 days, p.o.	Increased 5-HT and DA levels	Improvement in open field and water maze tests
	Mishra et al., 2021	42 (6/group)	Male Swiss mice	10, 15, 25, or 40 mg/Kg, i.p.	-	Dose dependent improvement in marble-burying behavior. No effects in motor activity

## Data Availability

Not applicable.

## Abbreviations

AchE	Acetylcholinesterase
ATP	Adenosine triphosphate
ASD	Autism spectrum disorders
Bcl	B-cell lympho MAO
BDI-II	Beck Depression Inventory II
BDNF	Brain-derived neurotrophic factor
BLYS	B lymphocyte stimulator
BZDs	Benzodiazepines
Ca^+2^	Calcium
cAMP	cyclic adenosine monophosphate
CAT	Catalase
CBR	Cannabinoid receptor
CCI	Chronic constriction injury
CDSS	Clinical depression screening scale
CGI-I	Clinical global impressions-improvement
CGI-S	Clinical global impressions-severity
CMS	Chronic mild stress
COX-2	Cyclooxygenase-2
CUMS	Chronic unpredictable mild stress
CORT	Corticosterone
CREB	cAMP response element-binding protein
CUS	Chronic unpredictable stress
CUR	Curcumin
CY-BOCS	Children’s Yale–Brown Obsessive Compulsive Scale
DA	Dopamine
DOPAC	4-dihydroxyphenylacetic acid
ECG	Electrocardiogram
EGFR	Epidermal growth factor receptor
ERK	Extracellular signal-regulated kinase
ET-1	Endothelin 1
GABA	Gamma-aminobutyric acid
GD	Gestational day
GDNF	Glial cell line-derived neurotrophic factor
GPx	Glutathione peroxidase
GSH	Glutathione
GST	Glutathione S-transferase
HADS	Hospital Anxiety and Depression Scale
HbA1c	Glycosylated hemoglobin A1c
HDL-C	High density lipoprotein cholesterol
HDRS	Hamilton Depression Rating Scale
HO-1	Heme oxygenase-1
ICAM-1	Intercellular adhesion molecule-1
IDO	Indolamine-2, 3-Dioxygenase
IDS-SR30	Inventory of depressive symptomatology
IFN-γ	Interferon
IL-1β	Interleukine-1β
IL-6	Interleukin-6
IL-10	Interleukin-10;
IMPS	Invalid metabolic panaceas
iNOS	Inducible nitric oxide synthase
IOS	Inflammation and oxidative stress
IRS-1	Insulin receptor substrate 1
LDL-C	Low density lipoprotein cholesterol
LOX-1	Lectin-like oxidized low-density lipoprotein receptor
Lp(a)	Lipoprotein(a)
LP	Lipooxigenase
LPS	Lipopolysaccharide
MADRS	Montgomery–Asberg Depression Rating Scale
MA	Motor activity test
MAO	Monoamine oxidase
MAPK	Mitogen-activated protein kinase
MBB	Marble-burying behavior test
MCAO	Middle cerebral artery occlusion
MDA	Malondialdehyde
MDD	Major depressive disorder
MEK	Methyl ethyl ketone
MMP	Mitochondrial membrane potential
MMP-9	Matrix metalloproteinase 9
mRNA	Messenger ribonucleic acid
NA	Noradrenaline
NAC	N-Acetylcysteine
NE	Norepinephrine
NF-κβ	Nuclear factor κβ
NGF	Nerve growth factor
NMDA	N-Methyl-D-Aspartate
NQO-1	Quinine oxidoreductase-1
OB	Olfactory bulbectomy
OCD	Obsessive compulsive disorder
OLS	Open label clinical studies
OVX	Ovarectomy/Ovarectomized
P38	P38 MAPK
PAINS	Pan assay interference compound
PANSS	Positive and negative syndrome scale
PGE-2	Prostaglandin E2
PET	Positron emission tomography
PKA	Protein kinase A
PKB	Protein kinase B
PLA2	Phospholipase A2
PPA	Propanoic acid
PPARγ	Peroxisome proliferator-activated receptor gamma
PSD-95	Postsynaptic density protein-95
RCT	Randomized clinical trial
sCAM-1	Soluble cell adhesion molecule 1
SCZ	Schizophrenia
SD	Sprague-Dawley
SL327	ERK inhibitor
SNRIs	Serotonin–norepinephrine reuptake inhibitors
SOD	Superoxide dismutase
SREBPs	Hepatic sterol regulatory element-binding proteins
SPS	Single prolonged stress
SSRIs	Selective serotonin reuptake inhibitors
STAI	State-Trait Anxiety Inventory
SUB-P	Substance P
TBARs	Thiobarbituric acid reactive substances
TBX-B2	Thromboxane B2
TCAs	Tricyclic antidepressants
TG	Triglycerides
TGF-β1	Transforming growth factor
TN	Trigeminal neuralgia
TNF-α	Tumor necrosis factor
Tuj1	Neuron-specific class III β-tubulin
VCAM-1	Vascular cell adhesion molecule-1
VEGF	Vascular endothelial growth factor
VPA	Valproic acid
YGTSS	Yale global tic severity scale
5-HIAA	High 5-hydroxyindoleacetic acid
5-HT	Serotonin

## Appendix A

Search terms used in this non-systematic review were: “curcumin and psychiatry”, “curcumin and neurology”, “curcumin and brain”, “curcumin and inflammation”, “curcumin and oxidative stress”, “curcumin and schizophrenia”, “turmeric and schizophrenia”, “curcumin and psychosis”, “curcumin and depression”, “curcumin and major depressive disorder”, “curcumin and MDD” “turmeric and depression”, “curcumin and autism”, “turmeric and autism”, “curcumin and obsessive-compulsive disorder”, “turmeric and obsessive-compulsive disorder”, “curcumin and OCD”.
